# Analytical methods for stable isotope labeling to elucidate rapid auxin kinetics in *Arabidopsis thaliana*

**DOI:** 10.1371/journal.pone.0303992

**Published:** 2024-05-22

**Authors:** Qian Tang, Molly Tillmann, Jerry D. Cohen

**Affiliations:** Department of Horticultural Science and Microbial and Plant Genomics Institute, University of Minnesota, Saint Paul, Minnesota, United States of America; Banaras Hindu University, INDIA

## Abstract

The phytohormone auxin plays a critical role in plant growth and development. Despite significant progress in elucidating metabolic pathways of the primary bioactive auxin, indole-3-acetic acid (IAA), over the past few decades, key components such as intermediates and enzymes have not been fully characterized, and the dynamic regulation of IAA metabolism in response to environmental signals has not been completely revealed. In this study, we established a protocol employing a highly sensitive liquid chromatography-mass spectrometry (LC-MS) instrumentation and a rapid stable isotope labeling approach. We treated *Arabidopsis* seedlings with two stable isotope labeled precursors ([^13^C_6_]anthranilate and [^13^C_8_, ^15^N_1_]indole) and monitored the label incorporation into proposed indolic compounds involved in IAA biosynthetic pathways. This Stable Isotope Labeled Kinetics (SILK) method allowed us to trace the turnover rates of IAA pathway precursors and product concurrently with a time scale of seconds to minutes. By measuring the entire pathways over time and using different isotopic tracer techniques, we demonstrated that these methods offer more detailed information about this complex interacting network of IAA biosynthesis, and should prove to be useful for studying auxin metabolic network in vivo in a variety of plant tissues and under different environmental conditions.

## Introduction

Plant growth control is regulated by changes in the levels of active hormones, their transport to and away from the site of action and the sensitivity of plant tissues to changes in these concentrations [1]. In particular, auxin levels in plants appear to be controlled by several different metabolic pathways. This combination of metabolic regulatory mechanisms has been termed “auxin homeostasis” to highlight the expected high degree of control exerted by plants to maintain optimal growth rates. Auxin homeostatic regulation involves an interactive network of redundant pathways, the complexity of which has been shown by the application of a combination of molecular, genetic, and analytical approaches [2–5].

A major limitation of many studies of phytohormone biosynthesis is the lack of dynamic information to allow interpretation of data in the context of metabolic fluxes. Metabolic analysis may demonstrate an increased abundance of a hormone or its metabolite under specific conditions. Often such studies cannot determine whether this is the result of increased flux from a biosynthetic enzyme, decreased flux by a catabolic enzyme, changes in formation or hydrolysis of conjugating moieties, or alteration in transport of the metabolite [6]. Furthermore, phytohormone metabolic pathways are largely interconnected, and a number of interacting metabolic processes maintain metabolite levels. These cannot be adequately resolved by measurement of steady-state metabolite concentrations alone.

These considerations are of concern because the vast bulk of studies investigating phytohormone metabolism have approached physiological questions by measuring hormone levels and/or metabolite levels to infer a metabolic origin, fate, or regulation, with only a few notable exceptions. Although many of these studies have been informative, they employ limited static “windows” that are often extrapolated to predict time-dependent metabolic processes. The full limitations of such approaches are difficult to predict with any certitude. Similarly, approaches that seek to measure reactions where a supplied metabolic precursor is applied and its conversions to a product of interest, typically using an isotopic label, provide different information but also are subject to some inherent limitations. These can be compounded by measurements that are limited in number of time points. Also, often measurements are made after protracted labeling periods. These times can range from somewhat less than an hour to several hours [7–13]. In other cases, labeling times can continue for a day to even weeks [14–16]. Clearly, temporal understanding can be a concern given that indole-3-acetic acid (IAA) turnover have been measured or modeled to be in the range of a few minutes to 1–10 hours [17–19].

To overcome some of the possible limitations of prior efforts, we developed a method for the targeted metabolomic analysis of the indole auxin biosynthetic network [20]. We have now further adapted this method by first analyzing when seedlings of *Arabidopsis* make the transition from heterotrophic to autotrophic auxin biosynthesis, and then by using seedlings of *Arabidopsis* to analyze the incorporation at stable isotopic labels into newly synthesized IAA as well as potential biochemical precursors at very short time intervals with time-dependent sampling. By these small molecule implementations of Stable Isotope Labeled Kinetics (SILK) methods, we demonstrate that measuring entire pathways over time and using different isotopic tracer precursor techniques offer improved and more detailed information about this complex interacting network of metabolic reactions.

## Materials and methods

### Growing plant material

Wild-type Columbia-0 *Arabidopsis* seeds were grown on [^15^N]*Arabidopsis thaliana* salts (ATS) media [21] containing K^15^NO_3_ (Cambridge Isotope Laboratories, NLM-765-PK) and Ca(^15^NO_3_)_2_ (Cambridge Isotope Laboratories, NLM-499-PK) salts with 1% agar (Phytotech, A111) and 1% sucrose (Millipore Sigma S5390). When required for labeling and inhibitor studies, 20 μm nylon mesh (Sefar, 03-20/14) were cut into 9 cm × 9 cm squares, autoclaved to sterilize for 45 minutes at 121°C, then placed flat onto [^15^N]ATS germination media in 10 cm × 10 cm square Petri dishes (Fisherbrand, FB0875711A). *Arabidopsis* seeds were surface sterilized by soaking in dilute bleach solution (20% commercial bleach (Clorox, 6% sodium hypochlorite), 80 mL deionized water, 20 μL Tween 80 (Sigma-Aldrich, P1754)) for 5 minutes and then rinsing 4 times with sterile water. Plates were stored in the cold room at 4°C in the dark for 3 days to stratify seeds, then removed from cold and placed vertically in growth chamber (10/14-h photoperiod, ~100 μmol m^-2^ s^-1^ white light at 22°C).

### Harvest, homogenization and extraction

These steps were performed as detailed below in two separate options. In each case, plant samples were thoroughly homogenized (with added internal standard, if desired, for quantitative analysis) and equilibrated for a short time before centrifugation to remove large particles.

#### Option 1a: Absolute quantification of unlabeled IAA and [^15^N_1_]IAA

[^13^C_6_]IAA (CAS 100849-36-3; IAA (phenyl-^13^C₆), 99atom%, Cambridge Isotope Laboratories, CLM-1896) was used as internal standard to find the change from the heterotrophic IAA utilization from stored reserves and precursors to autotrophic *de novo* IAA production. 5–20 mg of seedlings were collected into a 1.5 mL microcentrifuge tube (Fisherbrand, 05-408-129) every day at the same time from day 1 to day 14 after the transfer to the growth chamber. Microcentrifuge tubes were weighed before and after sample collection using an analytical balance to record sample fresh weight. Immediately after weighing, tubes were submerged in liquid nitrogen to flash freeze and placed on dry ice. Samples were stored at -80°C until extraction. 20 μL of homogenization buffer per 10 mg tissue and 2–3 stainless steel beads (1.6 mm diameter, Next Advance, SSB16) were added to each sample with 10 ng of [^13^C_6_]IAA mixed per 1 mL homogenization buffer (65% isopropanol, 35% 0.2 M imidazole (pH 7.0)). Samples were homogenized in Geno/Grinder (SPEX SamplePrep) for 4 minutes at 1750 RPM, followed by incubating on ice for approximately 1 hour. 90 μL of water were added to each homogenized sample per 10 μL homogenization buffer. After mixing, samples were centrifuged at 25,000 g for 10 min. at 4°C.

#### Option 1b: Extraction of IAA

IAA is generally present in plant tissues at low concentrations. The purification of IAA samples involvs a two-step process of solid phase extraction (SPE) with an amino (NH_2_) resin followed by a subsequent step using polymethylmethacrylate epoxide (PMME) resin. For the first step, ion exchange TopTips (Glygen, TT2EMT) were prepared with Bondesil-NH_2_ resin (Agilent, 12213020; suspended in water, 1:4 w:v) for SPE according to Liu et al. [22]. Each TopTip was prepared with 20 μL resin suspension, then washed with 50 μL each: hexane, acetonitrile, ethyl acetate; finally conditioned with 50 μL 0.2 M imidazole (pH 7.0) followed by 2 × 100 μL water. Supernatant was loaded onto prepared TopTips. For larger samples, 250 μL of supernatant can be loaded at a time and spun through. TopTips were washed with 50 μL methanol. Tubes under TopTip adapters were exchanged to fresh 2 mL tubes (Fisherbrand, 02-681-343) for elution with 3 × 50 μL of 0.25% phosphoric acid (PA), followed by adding 25 μL of 0.1 M succinic acid, pH 6.0 (SA) to each sample. For the second step, polymeric sorbent TopTips were prepared with 75 μL PMME resin (Macro-prep epoxide support resin (Bio-Rad, 156–0000), suspended in 0.1 M sodium bicarbonate (pH 7.0), 1:4 w:v), washed with 2 × 100 μL methanol and conditioned with 2 × 100 μL 5:1 PA:SA. Tubes under TopTip adapters were exchanged to clean 1.5 mL microcentrifuge tubes for elution with 2 × 50 μL methanol. Volume of each sample was reduced to approximately 20 μL with vacuum concentrator (SpeedVac, Savant; about 8 minutes). Samples were then moved to LC autosampler vial inserts for LC-MS analysis.

#### Option 2a: Labeling and inhibitor studies (Fig 1)

12-day seedlings were transferred onto inhibitor media (see S1 Table) or to control (mock) media to begin auxin biosynthesis studies. Mesh with seedlings from germination plates was gently lifted and lay flat onto [^15^N]ATS media containing 100 μM 5-[2,6-difluorophenyl]-2,4-dihydro-[1,2,4]-triazole-3-thione (YDF, [23]; CAS# 1094690-87-5, custom synthesis by LabSeeker, Wujiang City, China; dissolved in dimethyl sulfoxide (DMSO) and filter sterilized before adding to media), 30 μM 3,4-dichloro-α-[(1,3-dihydro-1,3-dioxo-2H-isoindol-2-yl)oxy]-benzene propanoic acid, methyl ester (PVM2153/KOK2153, [24]; CAS# 1394950-62-9, LabSeeker; dissolved in acetonitrile and filter sterilized), 50 μM [4-[(2-aminophenyl) sulfanyl]butyl] phosphonic acid (I26, [25, 26]; CAS# 191411-61-7, Chemspace US, Monmouth Jct, NJ; dissolved in DMSO and filter sterilized), or solvent mock treatment. Plates were covered and placed vertically into the growth chamber for 20–30 hours. Isotopic labeling treatments were started by flooding plates with 3 mL of 500 μM [^13^C_6_]anthranilate (Sigma-Aldrich,709530) or 500 μM [^13^C_8_,^15^N_1_]indole (Cambridge Isotope Laboratories, CNLM-4786-0). Plates were covered and placed flat under growth conditions. Samples were collected by gathering 20–50 mg of plant tissue at time intervals range from 30 seconds to 256 minutes, gently blotting away moisture on a KimWipe (Kimberly-Clark, KC34155EXL), and placing in a 1.5 mL microcentrifuge tube then flash freezing in liquid nitrogen. IAA extraction and quantification are similar to the steps for *de novo* IAA analysis described in Option 1b except the internal standard mixed in homogenization buffer was [^2^H_4_]IAA (a gift from R.S. Bandurski; [27]).

**Fig 1:**
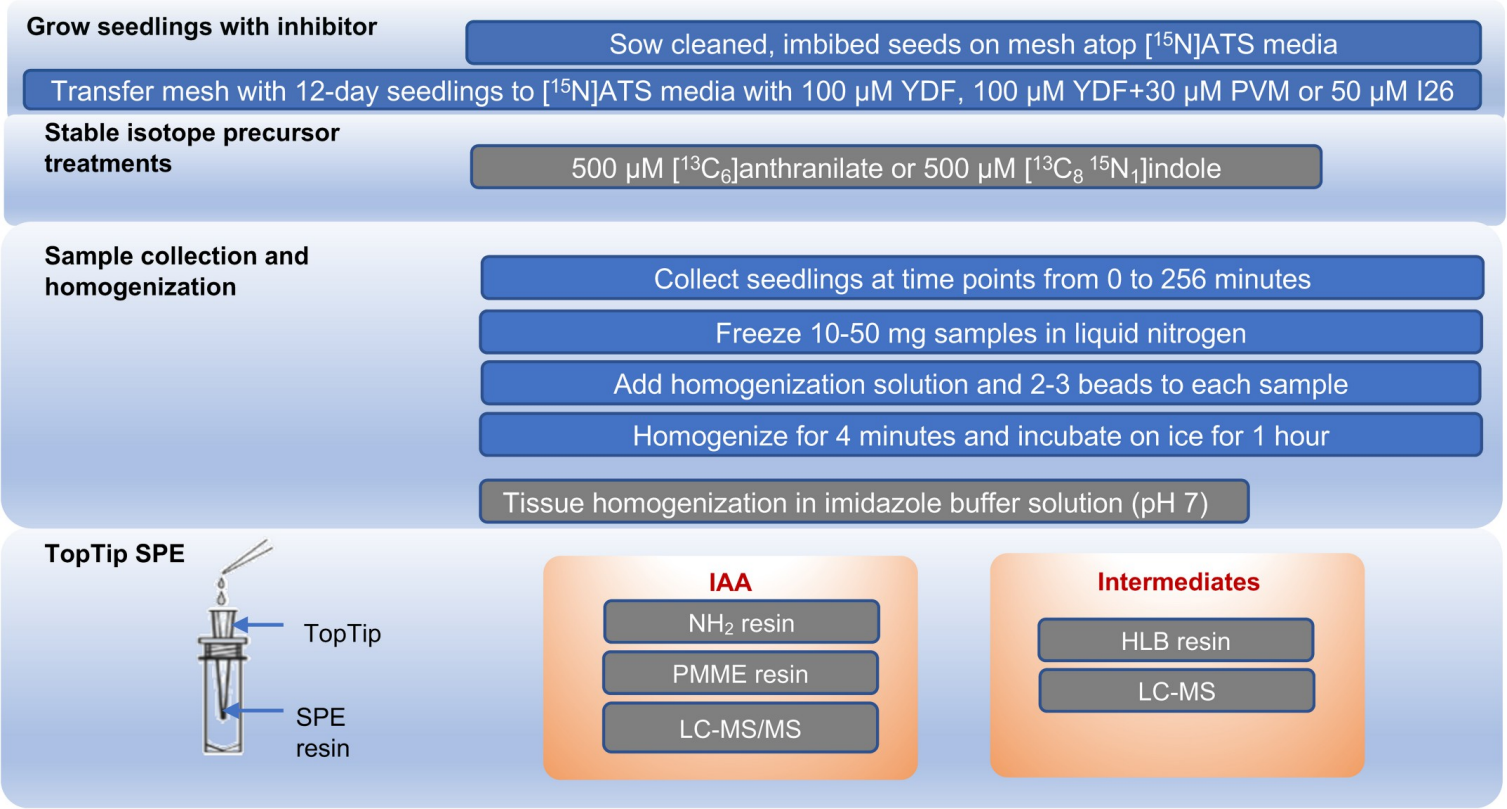
Stable isotope labeled kinetics (SILK) methods for analyzing IAA biosynthesis.

#### Option 2b: Extraction of proposed IAA biosynthesis intermediates: Anthranilate, IAAld, IPyA, IAOx, IAN, IAM, indole

For reverse isotope dilution quantitation [28], use unlabeled compounds as internal standards to quantify both endogenous compounds ([^15^N]-labeled in plants germinated on [^15^N]ATS media) and newly synthesized compounds (derived from a different isotopic form of labeled precursors (such as [^13^C_6_]anthranilate). 50 nM ANT (Sigma-Aldrich, A89855), 500 nM indole (Sigma-Aldrich, I0750), 5 μM Trp (Sigma-Aldrich, T8941), 1 nM IAM (Sigma-Aldrich, I1125), 2.5 μM IAN (Sigma-Aldrich, 129453), 100 nM IPyA (Sigma-Aldrich, I7017), 10 nM IAAld (Sigma-Aldrich, I1000), 10 nM IAA (Sigma-Aldrich, I3750), 1 nM TAM (Sigma-Aldrich, 193747), 10 nM IAOx (synthesized as described by Ahmad [29]) and 100 mM freshly prepared methoxylamine hydrochloride (CH_3_ONH_2_ ∙ HCl; Sigma-Aldrich, 226904; see [20]) were mixed into homogenization buffer. Samples were purified for analysis of IAA intermediates by SPE using a hydrophilic-lipophilic balanced (HLB) resin. IAA could alternatively be extracted using the following method, however, it may result in some loss of sensitivity compared to methods described in Option 1b. TopTips were prepared with 25 μL RENSA HLB resin ((MIP Technologies, 92001–0010) suspended in methanol, 1:5 w:v) and equilibrated with 2 × 50 μL 100% acetonitrile and 2 × 50 μL 20% acetonitrile. For highly sensitive detection and quantification of IAA, 200–300 μL of supernatant could be saved for separate IAA analysis (see Option 1b). Simultaneous extraction of IAA with precursor compounds may be conducted in samples containing sufficiently high IAA levels, typically ≥ 2 ng. After loaded with supernatant, TopTips were washed with 50 μL 5% acetonitrile. Tubes under TopTip adapters were exchanged to clean 1.5 mL tubes for elution with 2 × 50 μL 80% acetonitrile. Volume of each sample was reduced to approximately 20 μL using SpeedVac (about 10–12 minutes).

### LC-MS analysis

Samples were analyzed using ultra-high-performance liquid chromatography (UHPLC)-high resolution acurate mass (HRAM)-MS (Dionex UltiMate 3000 UHPLC, Q Exactive hybrid quadrupole/Orbitrap mass spectrometer, Xcalibur software (Thermo Scientific)) to chromatographically separate components of chemical matrix and obtain high resolution *m/z* (mass-to-charge ratio) data. In order to obtain highest sensitivity for the compounds of IAA biosynthesis, we chose a column (C_18_ HPLC column, 50 × 2.1 mm (Force, 9634252, Restek) with 0.2 μm precolumn filter (UltraShield, 25809, Restek)) with an end-capped octadecylsilane fully porous 1.8 μm silica resin with high carbon loading (20%). 5–10 μL of sample was injected for LC-MS analysis with mobile phase A, 0.1% formic acid in water (Optima™ LC/MS Grade, 7732-18-5 Fisher scientific) and B, 0.1% formic acid in acetonitrile. Different LC-MS methods were used to target compounds of interest. For IAA analysis, mobile phase gradient was 5% B (-1-0 min), 5–20% B (0–3 min), 20–80% B (3–6 min), 80% B (6–6.5 min) at a flow rate of 0.4 mL•min^-1^. Mass spectra were collected in positive ion mode in a parallel reaction monitoring (PRM) mode. The inclusion list contained ions of 176.1000, 177.1000, 180.1000, and 182.1000 *m/z*. PRM settings were: resolution: 17500 full width at half maximum (FWHM), automatic gain control (AGC) target: 2×10^5^, maximum ionization time: 50 milliseconds (ms), isolation window: 2.0 *m/z*, normalized collision energy (NCE): 20. Ion source settings were: spray voltage: 4.00 kV, capillary temperature: 275°C, probe heater temperature: 300°C, sheath gas: 30 arbitrary units, aux gas: 20 arbitrary units, S-lens RF level: 50. For analysis of all the proposed intermediates, plant extract were injected into the LC system and elution was with the following mobile phase gradient: 5% B (-2-1 min), 5–15% B (1–3 min), 15–30% B (3–3.5 min), 30% B (3.5–5 min), 30–39% B (5–7.5 min), 39–80% B (7.5–8 min), 80% B (8–8.5 min). The flow rate was 0.4 mL•min^-1^. Selected ion monitoring (SIM) mode was selected to collect mass spectra with a resolution of 70,000 FWHM, maximum ionization time of 200 ms and AGC of 5×10^5^. Ion source settings were: spray voltage: 4.00 kV, capillary temperature: 275°C, probe heater temperature: 300°C, sheath gas: 30 arbitrary units, aux gas: 20 arbitrary units, S-lens RF level: 50. The mass spectrometer was set to acquire several segments of full SIM scans, each targeting 1–3 compounds. The segments were: 200–217 *m/z* (0–2.1 min), 157–173 *m/z* (2.1–3 min), 133–150 *m/z* (3–3.74 min), 170–188 *m/z* (3.74–5.4 min), 152–170 *m/z* (5.4–6 min), 227–245 *m/z* (6–6.7 min), 184–201 *m/z* (6.7–8.5 min).

### Data analysis

#### IAA analysis

Extracted ion chromatograms (EICs) of labeled and unlabeled quinolinium ions resulting from the fragmentation of labeled internal standard and endogenous IAA were observed. Narrow mass ranges were used to filter out background noise. Under the “Ranges” tab in “Chromatogram Ranges” in Xcalibur, the chromatogram viewing options were set to display three mass ranges for *de novo* IAA analysis: 130.0641–130.0661 (corresponding to unlabeled quinolinium ion), 131.0612–131.0632 (corresponding to [^15^N_1_]quinolinium ion from autotrophic IAA production) and 136.0843–136.0863 ([^13^C_6_]quinolinium ion produced from [^13^C_6_]IAA internal standard); and four mass ranges for IAA pathway analysis: 131.0612–131.0632 (corresponding to [^15^N_1_]quinolinium ion from endogenous IAA produced in [^15^N]ATS media), 134.0892–134.0912(corresponding to [^2^H_4_] quinolinium ion from [^2^H_4_]IAA internal standard), and 136.0843–136.0863 ([^13^C_6_]quinolinium ion produced from [^13^C_6_]IAA after stable isotope labeling with [^13^C_6_]anthranilate) or 139.0880–139.0900 ([^13^C_8_,^15^N_1_]quinolinium ion produced from [^13^C_8_,^15^N_1_]IAA after stable isotope labeling with [^13^C_8_,^15^N_1_]indole). Under “Display” tab, “Peak Area” option was checked, followed by using the “peak selection” tool to select and calculate area of peaks corresponding to unlabeled or labeled IAA and the internal standard. Endogenous and labeled IAA levels were calculated using isotope dilution [30].

#### IAA biosynthesis intermediates analysis

A script was employed to determine peak areas from EICs of multiple compounds. Narrow mass ranges centered around the exact masses of ions generated by the compounds of interest, along with their labeled counterparts synthesized from the supplied labeled precursors, were defined to minimize background noise. Before inputting into R, raw data files were converted to *mzXML* format using the *msConvert* tool from the ProteoWizard software [31]. Quantitative data for each indolic compound of interest was extracted using the Metabolite-Turnover script developed in the Hegeman lab. (https://github.com/HegemanLab/Metabolite-Turnover, [32]). Within this script, the *ProteinTurnover* [33] and the *XCMS* package [34] are utilized to extract EICs for every isotopomer of IAA and intermediates. This quantification approach employing linear regression [35] is preferred over peak area-based quantification [36] when the MS data exhibits high background noise due to low analyte abundance. Exact masses for isotopomers of proposed IAA biosynthetic intermediates were calculated using the University of Wisconsin—Madison Biological Magnetic Resonance Data Bank exact mass calculator (http://www.bmrb.wisc.edu/metabolomics/mol_mass.php). These isotopomers of interest, derived from various isotopic labeling strategies, are listed in Tillmann et al. [20]. In the.CSV data output files, the slope of each linear regression line denotes the ratio between the corresponding isotopic trace and its monoisotopomer. This ratio was used to calculate the relative abundance of labeled compounds, enabling us to monitor the label incorporation from upstream precursors into IAA intermediates through multiple pathways.

## Results and discussion

An important advantage of SILK is that it can be used to assess not only the status on on-going metabolic activity but also the effect of various metabolic modulators on a target metabolic network [37]. The quantification of compounds as well as the rate of labeling has several advantages over static measurements of concentration [6]. For example, both metabolic activity and pathway disruptions are not adequately visualized by simply quantifying the concentrations before and after treatments. SILK, however, quantifies the metabolism of target compounds and can, for example, reflect which side of the biosynthesis/catabolism process has been affected and may reveal metabolism changes even if overall metabolite concentrations are essentially unchanged. In addition, SILK can be adapted, as employed in this method, to measure labeling patterns very rapidly in complex networks and determine changes in labeling as altered by metabolic disruptions over short time intervals [38].

**Fig 2:**
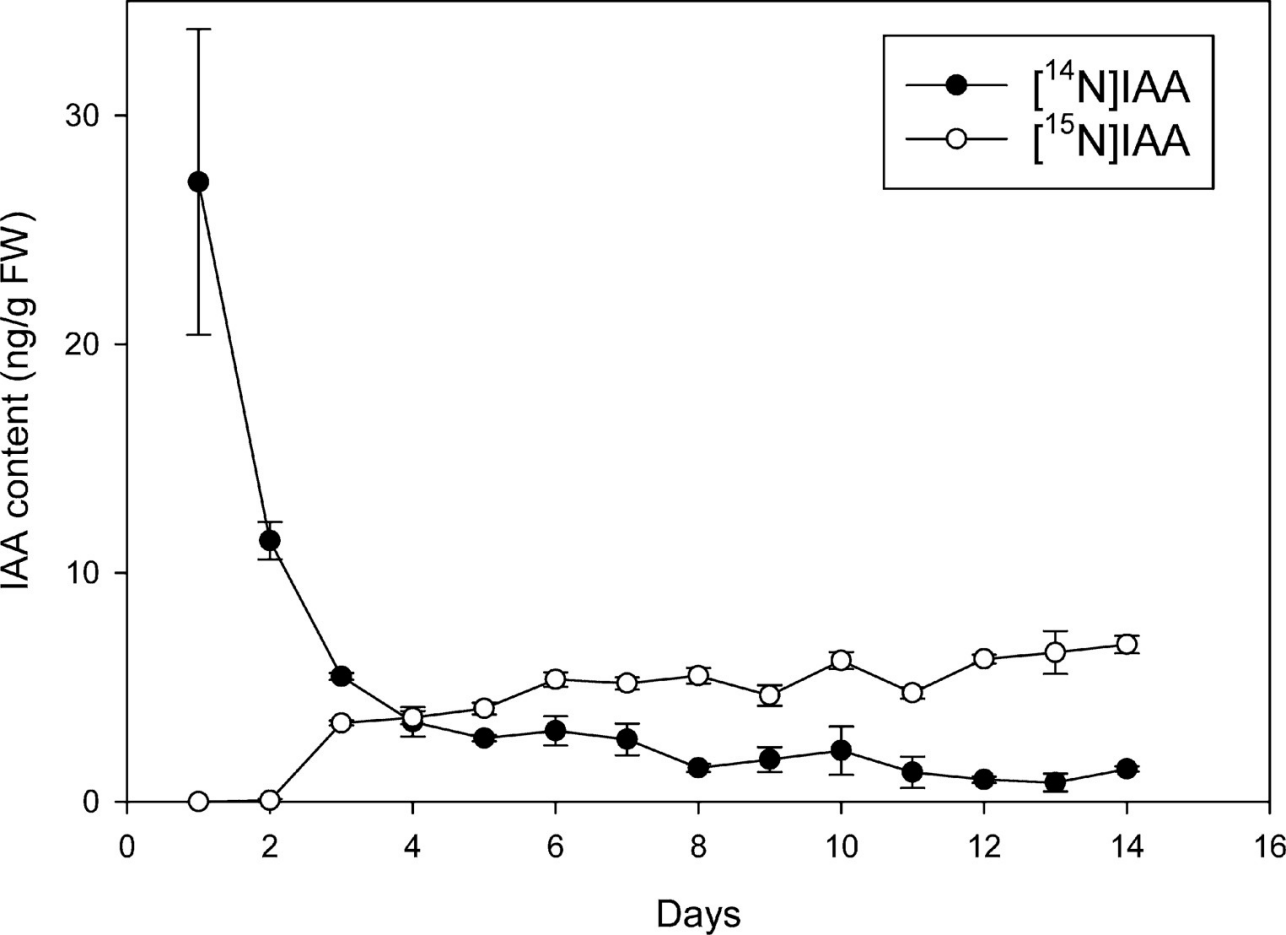
*De novo* IAA biosynthesis in *Arabidopsis* seed germination. Seeds were germinated on [^15^N] ATS media under 10/14-h photoperiod, 89 μmol m^-2^ s^-1^ white light at 22°C. 5–20 mg of seedlings were collected every day for 14 days followed by LC-MS/MS. Unlabeled and [^15^N_1_]IAA were quantified by isotope dilution using [^13^C_6_]IAA internal standard. Each point represents the mean of 5 biological replicates; error bars represent SE. [^14^N_1_]IAA and [^15^N_1_]IAA levels are significant different from day 12 (p<0.05; Student’s t test).

For the analysis of IAA biosynthesis in a seedling it is better defined after the plant has become fully autotrophic for its auxin needs; this limits inputs from large stores of storage forms [39]. There are only a few studies in *Arabidopsis* that have accessed this critical transition period, although a few reports exist employing deuterium oxide with plants with larger seeds such a maize [40–43] and bean [44]. These prior studies utilized deuterium oxide primarily because of its rapid uptake into cellular compartments and because the low-resolution mass spectrometers available at the time were better suited to analyze multiple labels to avoid interference with naturally occurring heavy atoms, primarily endogenous ^13^C, that gave IAA approximately a 11.1% *m+1/z* increase in the isotopic envelope. The growth of plants on deuterium oxide however was shown to be inhibited [44, 45] thus making evaluation of temporal changes difficult to reconcile. In addition, deuterium exchange processes [27, 40, 44] complicates isotopic analysis. Modern high resolution mass spectrometers, however, can easily distinguish between a mass increase of 1.00335 for ^13^C, 1.00628 for ^2^H and a mass increase of 0.99703 for ^15^N, allowing the structural nitrogen atom to serve as a monitor of new synthesis. Methods for growth with ^15^N have been developed [20]. Structural atoms such as ^15^N, ^18^O and ^13^C avoid many of the problems of exchangeable deuterium, as high concentrations are not toxic, nor do they alter growth rates, and they are not generally subject to rearrangements [46] during mass spectrometry processes. One disadvantage to using inorganic ^15^N to measure *de novo* synthesis of a nitrogen-containing molecule is the recycling of nitrogen that occurs [47], however that change in the baseline of enrichment becomes important only where absolute rates of synthesis are being measured. As shown in Fig 2, low levels of ^14^N become consistent at day 8, remains low, and “new IAA” with the [^15^N]-label becomes the predominate form. Our labeling experiments were thus done with 12-day-old seedlings so they are well beyond the “auxin autotropic” stage based on [^15^N]-incorporation.

Previous studies using isotopic labeling measured label incorporation after hours (3 or 6 hrs, [10]; 6, 10, 21 hrs, [48]) or days (3 or 6 days, [44]; 19 days, [14]) which has the potential to complicate data interpretation for a complex pathway where rates of reactions are potentially much faster than the analysis times [18]. The goals of this current research were to develop a procedure where labeling times could be reduced, and the kinetics of labeling could be measured over relatively short time intervals.

**Fig 3:**
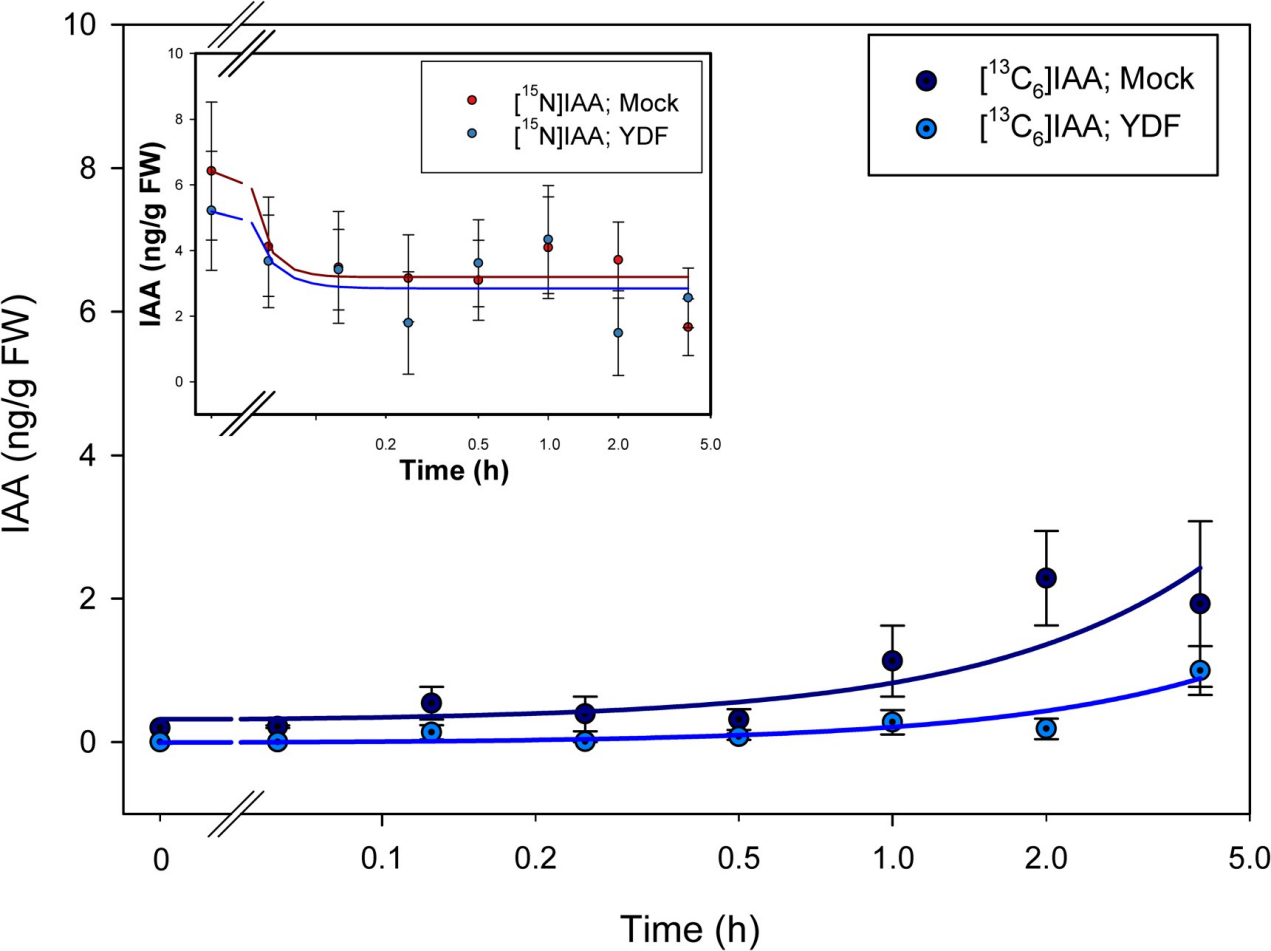
[^13^C_6_]anthranilate labeling patterns of IAA in *Arabidopsis* seedlings treated with YDF. Seedlings were grown on [^15^N] ATS media under 10/14-h photoperiod, 103 μmol m^-2^ s^-1^ white light at 22°C for 12 days, then transferred onto [^15^N] ATS media containing 100 μM YDF or DMSO (mock). After 20 hours seedlings were treated by applying, to the inhibitor or mock plates, ATS solution containing 500 μM [^13^C_6_]anthranilate. 20–30 mg of seedlings were collected at time points from 0–4 hours, then subject to LC-MS/MS analysis. [^15^N_1_]IAA and [^13^C_6_]IAA were quantified by isotope dilution using [^2^H_4_]IAA internal standard. Each point represents the mean of 3 biological replicates; error bars represent SE. Fitted lines were drawn using SigmaPlot 14.0 Dynamic Regression standard curve and four parameter procedures. [^13^C_6_]IAA levels are significantly different between mock and YDF treatment after 2 hours of incubation (p<0.05; Student’s t test).

**Fig 4:**
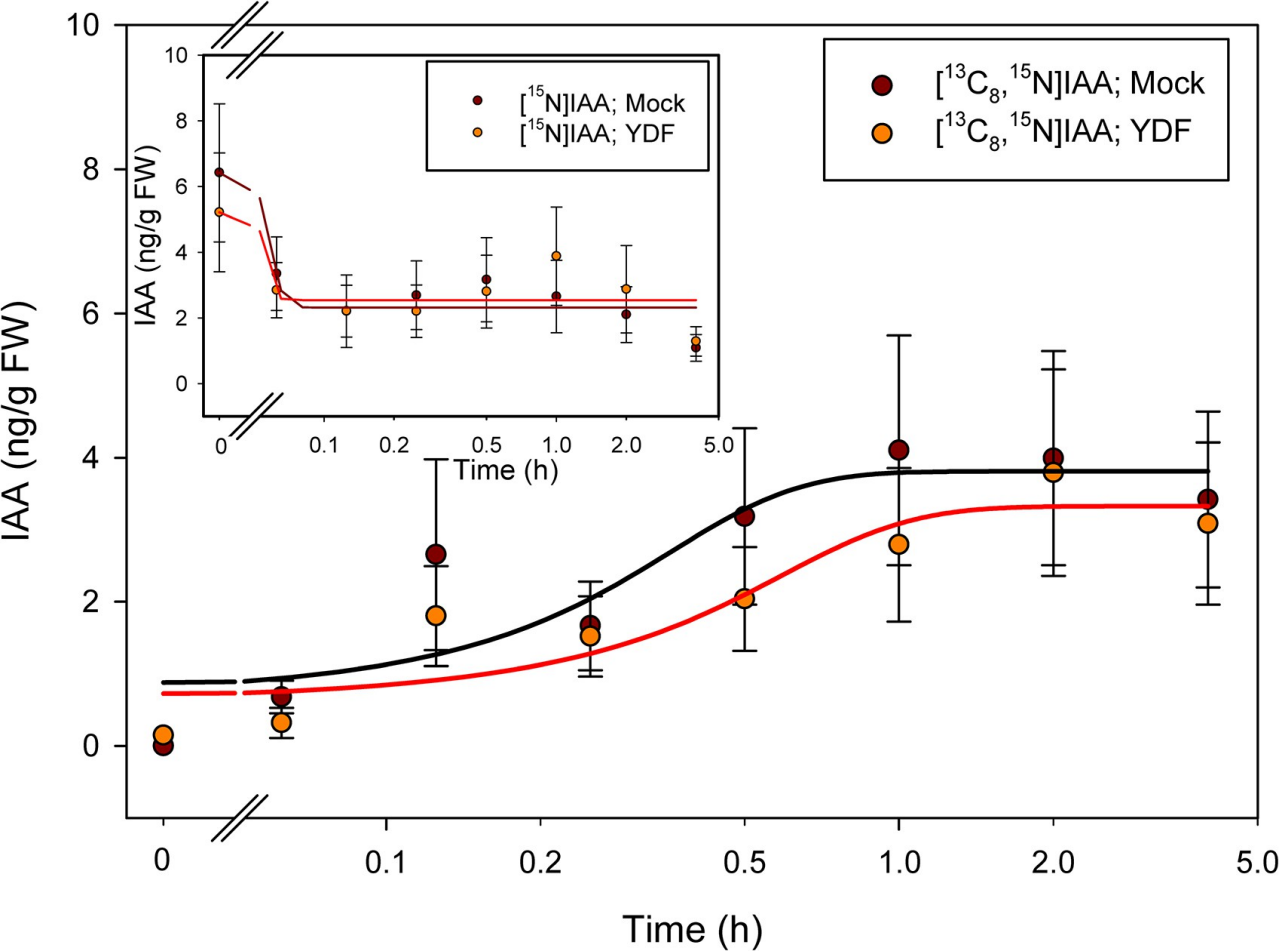
[^13^C_8_,^15^N_1_]indole labeling patterns of IAA in *Arabidopsis* seedlings treated with YDF. Seedlings were grown on [^15^N] ATS media under 10/14-h photoperiod, 103 μmol m^-2^ s^-1^ white light at 22°C for 12 days, then transferred onto [^15^N] ATS media containing 100 μM YDF or DMSO (mock). After 20 hours seedlings were treated by applying, to the inhibitor or mock plates, ATS solution containing 500 μM [^13^C_8_,^15^N_1_]indole. 20–30 mg of seedlings were collected at time points from 0–4 hours, then subject to LC-MS/MS analysis. [^15^N_1_]IAA and [^13^C_8_,^15^N_1_]IAA were quantified by isotope dilution using [^2^H_4_]IAA internal standard. Each point represents the mean of 3 biological replicates; error bars represent SE. Fitted lines were drawn using SigmaPlot 14.0 Dynamic Regression with sigmoid 3 and with four parameter procedures. [^13^C_8_,^15^N_1_]IAA levels are not significantly different between mock and YDF treatment across all time points (p>0.05; Student’s t test).

**Fig 5:**
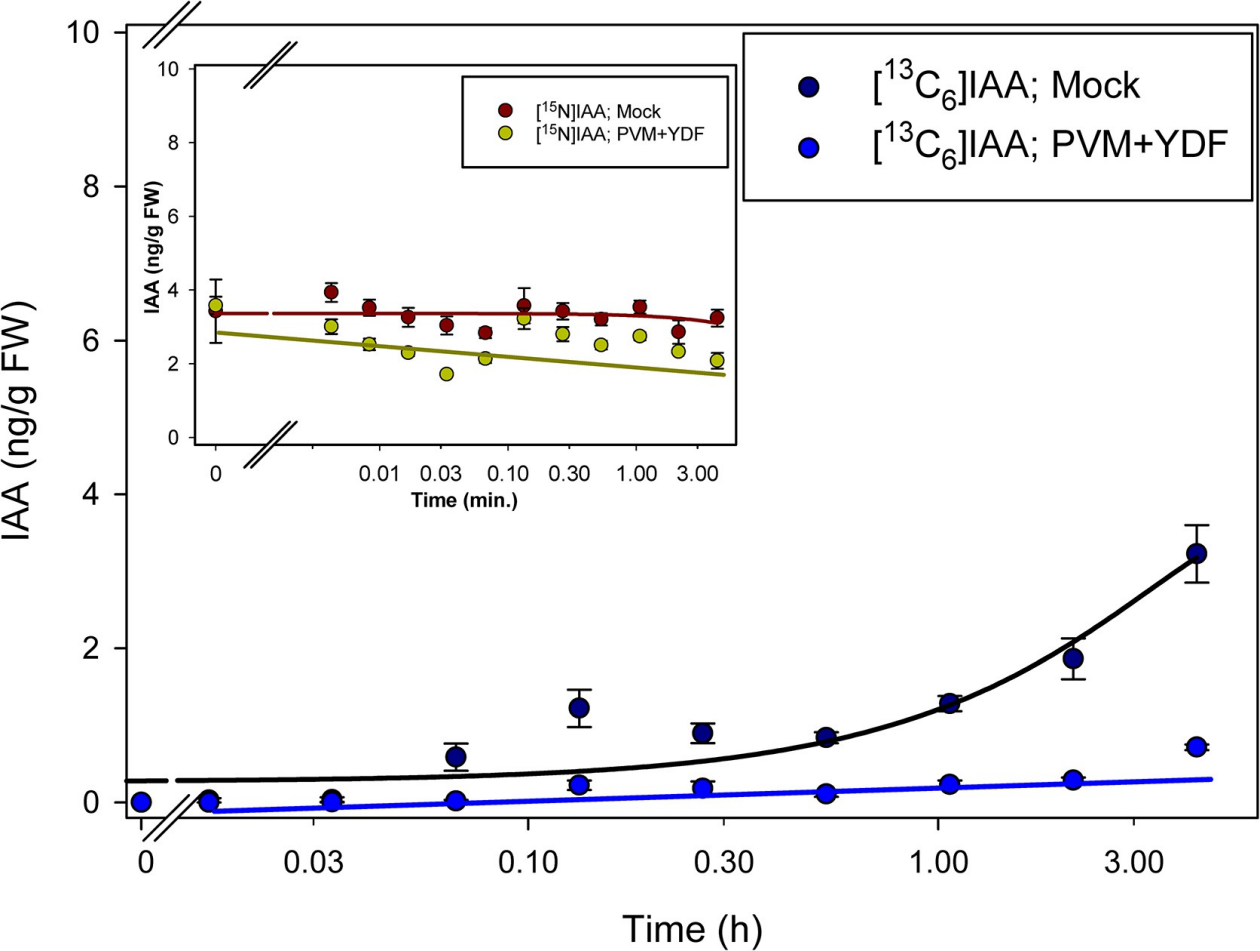
[^13^C_6_]anthranilate labeling patterns of IAA in *Arabidopsis* seedlings in the presence of IPyA pathway inhibitors. Seedlings were grown on [^15^N] ATS media under 10/14-h photoperiod, 103 μmol m^-2^ s^-1^ white light at 22°C for 12 days, then transferred onto [^15^N] ATS media containing 100 μM YDF and 30 μM PVM2153, or DMSO and acetonitrile (mock). After 20 hours seedlings were treated by applying, to the inhibitor or mock plates, ATS solution containing 500 μM [^13^C_6_]anthranilate. 20–30 mg of seedlings were collected at time points from 0–4.3 h (0 to 256 minutes), then subject to LC-MS/MS analysis. [^15^N_1_]IAA and [^13^C_6_]IAA were quantified by isotope dilution using [^2^H_4_]IAA internal standard. Fitted lines were drawn using SigmaPlot 14.0 Dynamic Regression standard curve procedures. [^13^C_6_]IAA levels are significantly different between mock and inhibitor treatment from 0.07 to 4.3 h (4 to 256) minutes of incubation (p<0.05; Student’s t test).

**Fig 6:**
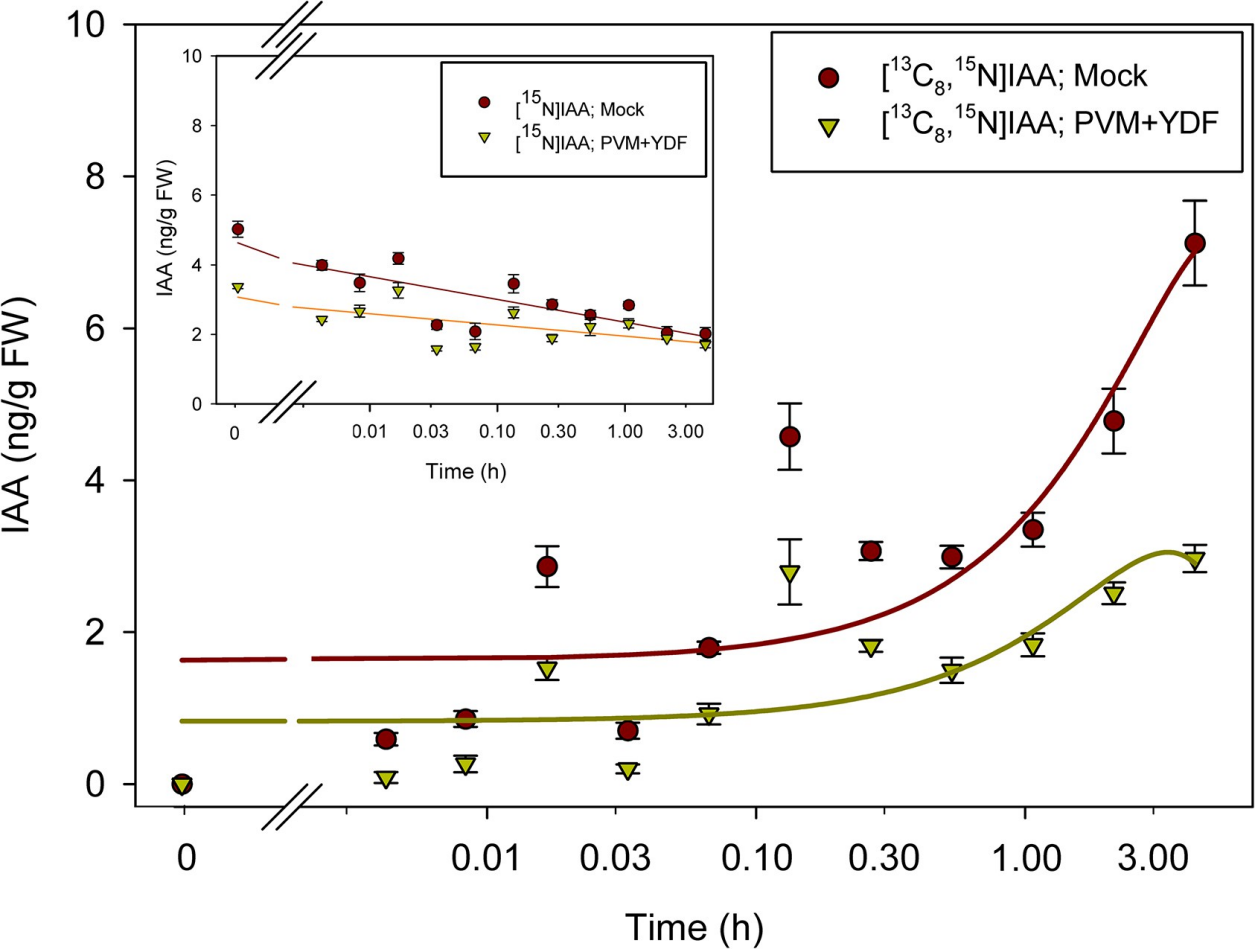
[^13^C_8_,^15^N_1_]indole labeling patterns of IAA in *Arabidopsis* seedlings in the presence of IPyA pathway inhibitors. Seedlings were grown on [^15^N] ATS media under 10/14-h photoperiod, 103 μmol m^-2^ s^-1^ white light at 22°C for 12 days, then transferred onto [^15^N] ATS media containing 100 μM YDF and 30 μM PVM2153, or DMSO and acetonitrile (mock). After 20 hours seedlings were treated by applying, to the inhibitor or mock plates, ATS solution containing 500 μM [^13^C_8_,^15^N_1_]indole. 20–30 mg of seedlings were collected at time points from 0–4.3 h (0 to 256 minutes), then subject to LC-MS/MS analysis. [^15^N_1_]IAA and [^13^C_8_,^15^N_1_]IAA were quantified by isotope dilution using [^2^H_4_]IAA internal standard. Fitted lines were drawn using SigmaPlot 14.0 Dynamic Regression standard curve procedures. [^13^C_8_,^15^N_1_]IAA levels are significantly different between mock and inhibitor treatment from 0.08 to 4.3 h (0.5 to 256 minutes) of incubation (p<0.05; Student’s t test).

By labeling plants with [^13^C_6_]anthranilate, the appearance of [^13^C_6_]IAA could be detected after 1 hour of incubation (Fig 3), with little or no change in the [^15^N_1_]IAA pool except for a decline immediately upon transfer to the label. After 2 hours of incubation, labeling from [^13^C_6_]anthranilate into IAA was reduced by treatment with the monooxygenase inhibitor YDF (Fig 3). Labeling with [^13^C_8_,^15^N_1_]indole was more rapid than labeling with [^13^C_6_]anthranilate, suggesting perhaps more rapid uptake as might be expected for a lipid soluble compound. Interestingly, YDF had little effect on the incorporation of label into [^13^C_8_,^15^N_1_]IAA (Fig 4). This is similar to what we previously reported after a longer-term incubation [49] but without time resolved data. The effect of IPyA pathway inhibition by both the tryptophan amino transferase inhibitor PVM2153 and the monooxygenase inhibitor YDF was more pronounced than YDF alone, with little incorporation of ^13^C into IAA from [^13^C_6_]anthranilate labeling even after 4 hours with both inhibitors (Fig 5). With [^13^C_8_,^15^N_1_]indole and both inhibitors, labeling of IAA was reduced more than with YDF alone, but still not as pronounced as with [^13^C_6_]anthranilate (Fig 6).

**Fig 7:**
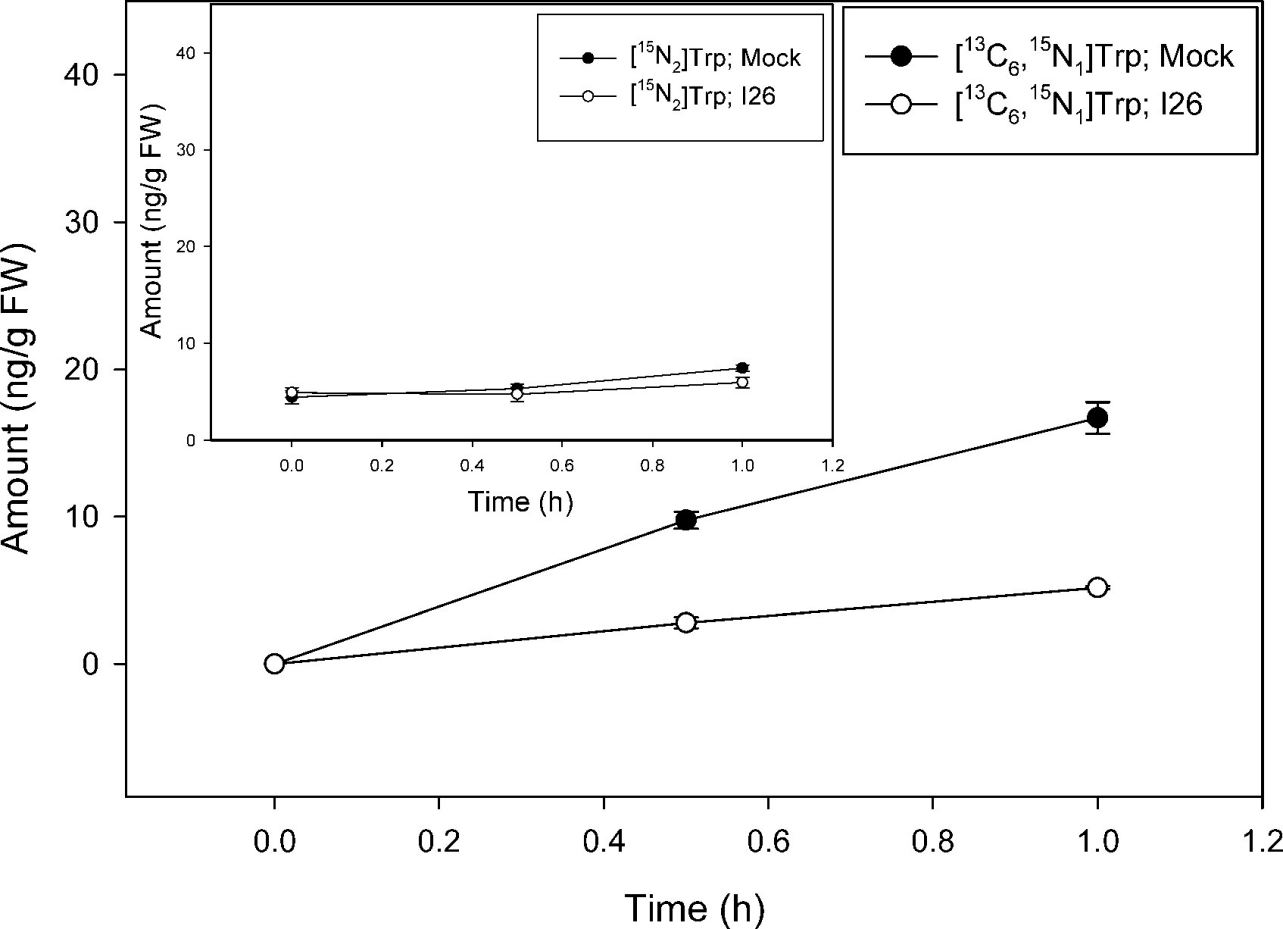
[^13^C_6_]anthranilate labeling patterns of Trp in *Arabidopsis* seedlings in the presence of TSα inhibitor compound 26 (arylsulfide phosphonate). Seedlings were grown on [^15^N] ATS media under 10/14-h photoperiod, 103 μmol m^-2^ s^-1^ white light at 22°C for 12 days, then transferred onto [^15^N] ATS media containing 50 μM compound 26 or DMSO (mock). After 23 hours seedlings were treated by applying, to the inhibitor or mock plates, ATS solution containing 500 μM [^13^C_6_]anthranilate. 20–30 mg of seedlings were collected at 0, 30 and 60 minutes, then subject to LC-MS analysis. [^15^N_2_]Trp and [^13^C_6,_^15^N_1_]Trp were quantified by using a linear regression approach employing the XCMS package in R. Each point represents the mean of 3 biological replicates; error bars represent SE. [^15^N_2_]Trp levels are significantly different between mock and I26 treatment after 1 hour of incubation and [^13^C_6_,^15^N_1_]Trp levels are significantly different between mock and I26 treatment after 0.5 and 1 hour of incubation (p<0.05; Student’s t test).

**Fig 8:**
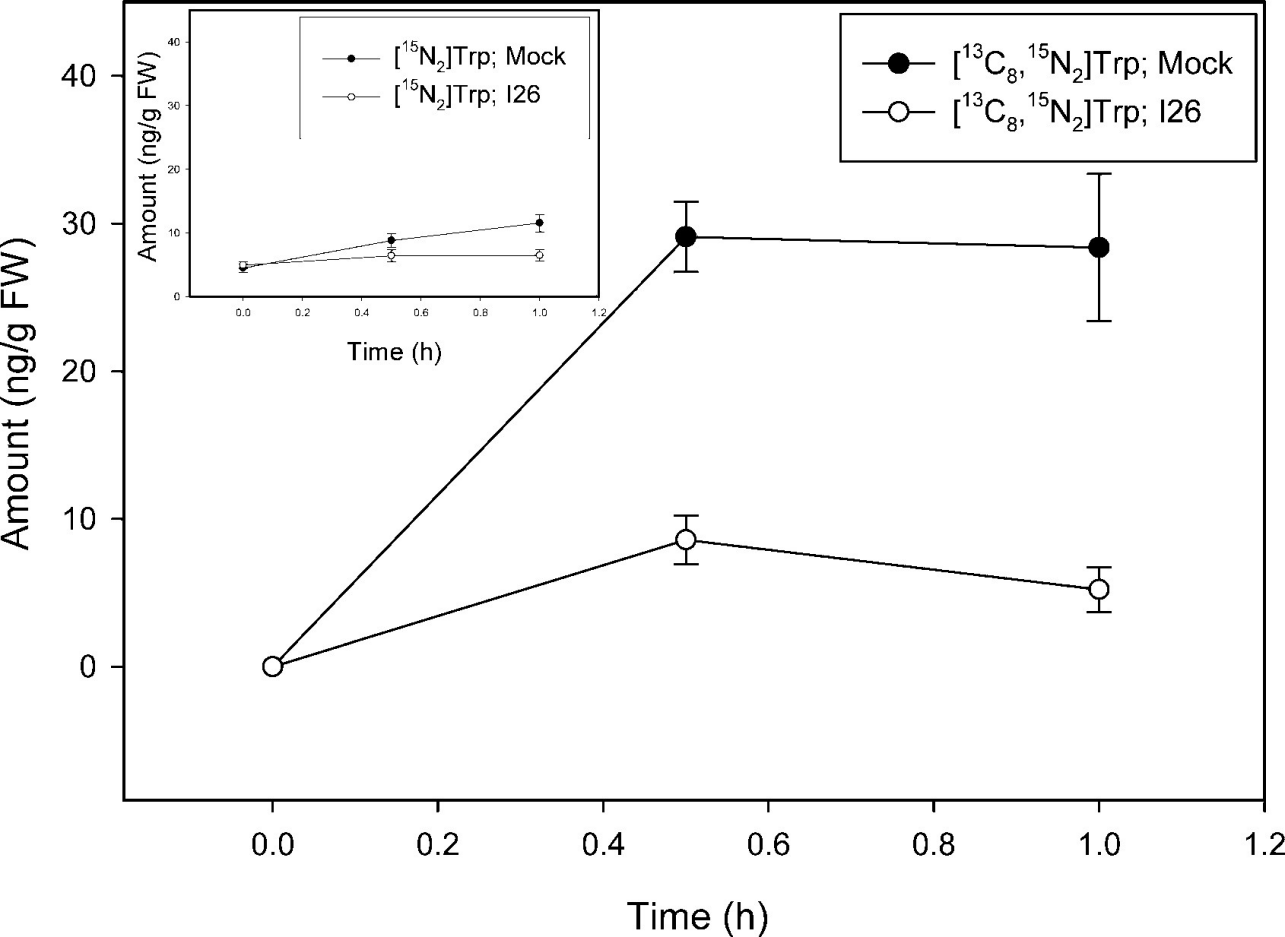
[^13^C_8_,^15^N_1_]indole labeling patterns of Trp in *Arabidopsis* seedlings in the presence of TSα inhibitor compound 26 (arylsulfide phosphonate). Seedlings were grown on [^15^N] ATS media under 10/14-h photoperiod, 103 μmol m^-2^ s^-1^ white light at 22°C for 12 days, then transferred onto [^15^N] ATS media containing 50 μM compound 26 or DMSO (mock). After 23 hours seedlings were treated by applying, to the inhibitor or mock plates, ATS solution containing 500 μM [^13^C_8_,^15^N_1_]indole. 20–30 mg of seedlings were collected at 0, 30 and 60 minutes, then subject to LC-MS analysis. [^15^N_2_]Trp and [^13^C_8_,^15^N_2_]Trp were quantified by using a linear regression approach employing the XCMS package in R. Each point represents the mean of 3 biological replicates; error bars represent SE. [^15^N_2_]Trp levels are significantly different between mock and I26 treatment after 1 hour of incubation and [^13^C_8_,^15^N_2_]Trp levels are significantly different between mock and I26 treatment after 0.5 and 1 hour of incubation (p<0.05; Student’s t test).

**Fig 9:**
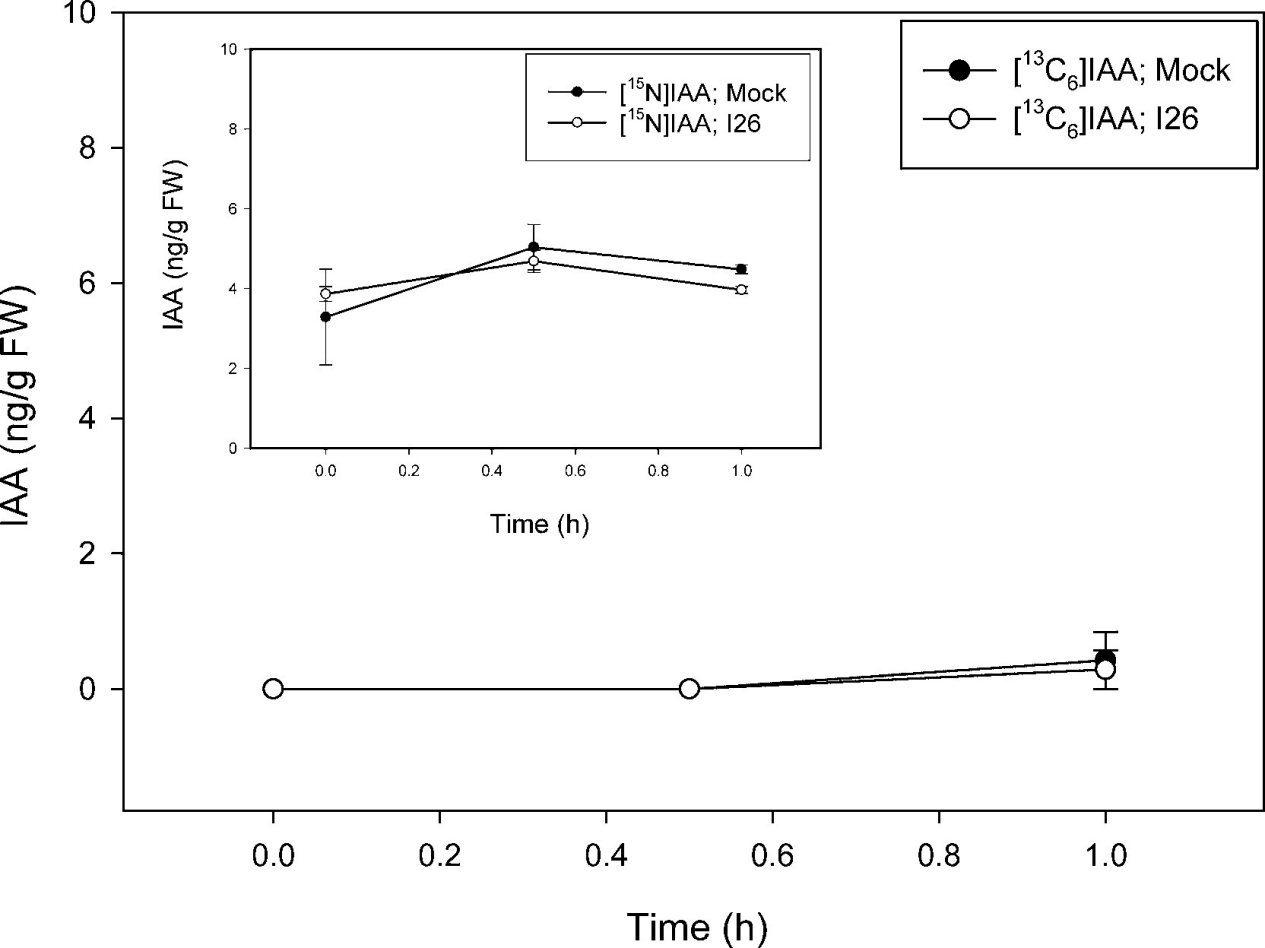
[^13^C_6_]anthranilate labeling patterns of IAA in *Arabidopsis* seedlings in the presence of TSα inhibitor compound 26 (arylsulfide phosphonate). Seedlings were grown on [^15^N] ATS media under 10/14-h photoperiod, 103 μmol m^-2^ s^-1^ white light at 22°C for 12 days, then transferred onto [^15^N] ATS media containing 50 μM compound 26 or DMSO (mock). After 23 hours seedlings were treated by applying, to the inhibitor or mock plates, ATS solution containing 500 μM [^13^C_6_]anthranilate. 20–30 mg of seedlings were collected at 0, 30 and 60 minutes, then subject to LC-MS analysis. [^15^N_1_]IAA and [^13^C_6_]IAA were quantified by isotope dilution using [^2^H_4_]IAA internal standard. Each point represents the mean of 3 biological replicates; error bars represent SE. [^13^C_6_]IAA levels are not significantly different between mock and I26 treatment after 0.5 and 1 hour of incubation (p>0.05; Student’s t test).

**Fig 10:**
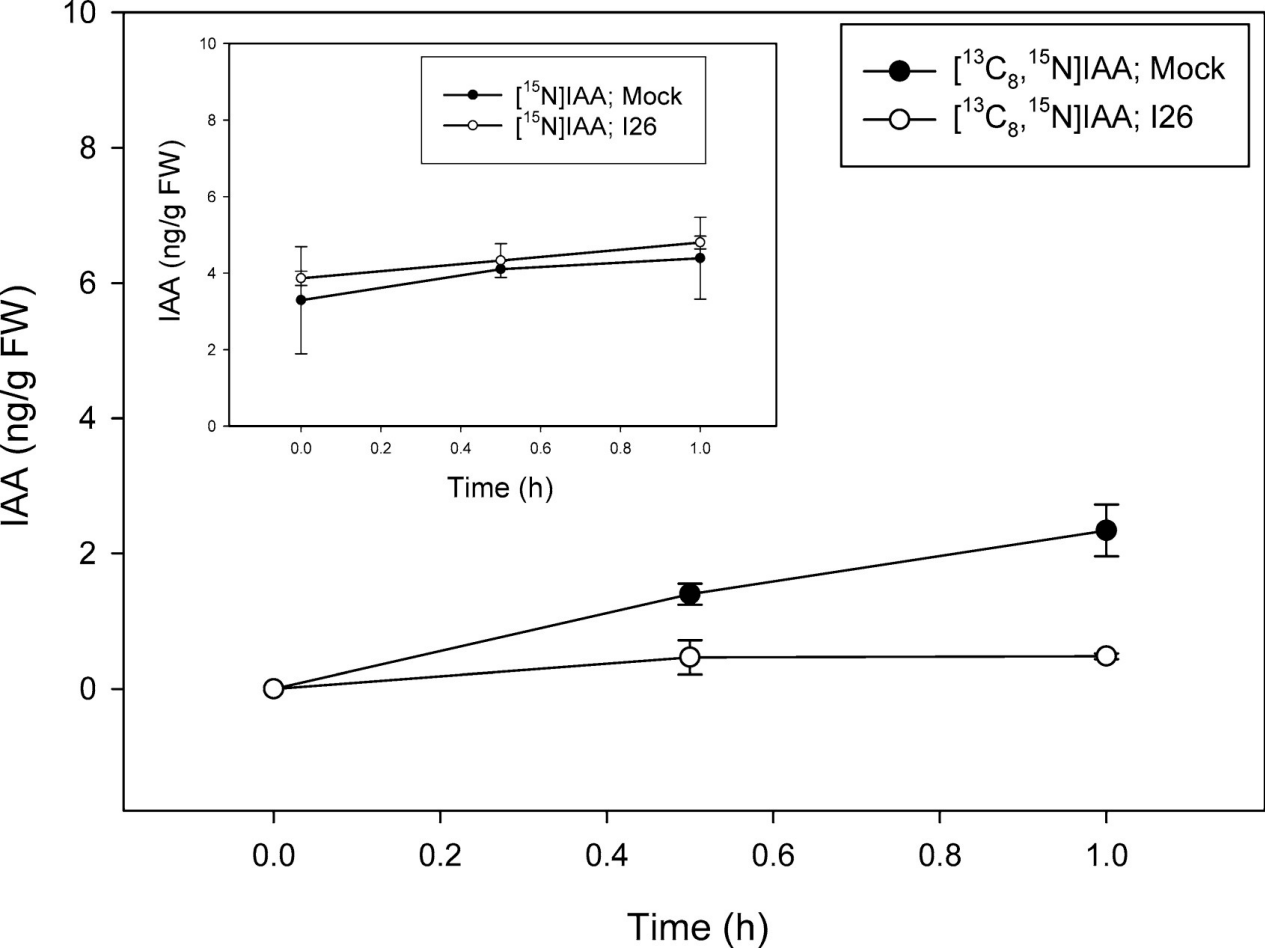
[^13^C_8_,^15^N_1_]indole labeling patterns of IAA in *Arabidopsis* seedlings in the presence of TSα inhibitor compound 26 (arylsulfide phosphonate). Seedlings were grown on [^15^N] ATS media under 10/14-h photoperiod, 103 μmol m^-2^ s^-1^ white light at 22°C for 12 days, then transferred onto [^15^N] ATS media containing 50 μM compound 26 or DMSO (mock). After 23 hours seedlings were treated by applying, to the inhibitor or mock plates, ATS solution containing 500 μM [^13^C_8_,^15^N_1_]indole. 20–30 mg of seedlings were collected at 0, 30 and 60 minutes, then subject to LC-MS analysis. [^15^N_1_]IAA and [^13^C_8_,^15^N_1_]IAA were quantified by isotope dilution using [^2^H_4_]IAA internal standard. Each point represents the mean of 3 biological replicates; error bars represent SE. [^13^C_8_,^15^N_1_]IAA levels are significantly different between mock and I26 treatment after 1 hour of incubation (p>0.05; Student’s t test).

Inhibition of the biosynthesis of tryptophan as well as reactions related to tryptophan is complex in plants in part because indolic compounds and derivatives are often accumulated as part of the biotic resistance system [50]. In addition to two gene copies encoding most of the enzymes involved in the conversion of anthranilate to tryptophan, including tryptophan synthase (TS) α and β subunits, *Arabidopsis* has the TSα-like indole synthase (INS), a type2 AtTSβ protein, and an undefined AtTSβtype1 [16, 51–54]. A number of different inhibitors are known for the canonical TSαββα heterotetramer [20], however, such metabolic modulators have not been explored as to how they alter the activities of INS, nor standalone type1 and type2 AtTSβ proteins. Nevertheless, compounds that inhibit the activity of these early steps related to IAA metabolism have promise for further analysis of the precursors involved in the pathways. In a proof-of-concept study we used a well-established inhibitor of TS, an arylsulfide phosphonate (I26; [4-[(2-aminophenyl) sulfanyl]butyl] phosphonic acid) that was originally designed as a transition state analog targeting TSα. TS inhibitors are, however, well known to exert pleiotropic effects across the TSαββα heterotetramer due to complex allosteric interactions and little is known about their effects on the TSα-like INS enzyme, AtTSβtype2 or AtTSβtype1 [52]. As shown in Fig 7, I26 essentially blocks new Trp synthesis from [^13^C_6_]anthranilate over a 23 h period with an inhibition on the slightly increasing levels of [^15^N_2_]Trp (Fig 7 insert) as would be predicted for a TSα inhibitor. The slight increase in [^15^N_2_]Trp pool is likely due to the continued dilution of ^14^N from plant internal pools (Fig 2, [49]). I26 also, however, markedly inhibits Trp synthesis from [^13^C_8_,^15^N_1_]indole (Fig 8) as well as from endogenously derived indole/serine to form [^15^N_2_]Trp (Fig 8 insert). This result is not expected based on the first reports on I26 and its predicted mechanism. Nevertheless because, as discussed in Michalska et al. [55], TSαβ is allosterically regulated by the physical switching of the α- and β-subunits between an open low activity confirmation and a closed high activity conformation, interaction between TSαβ can be complex. In their open conformations, active sites are freely accessible to supplied substrates while in their closed states, sites are inaccessible to external substrates. While this switching normally prevents the escape of the intermediate, indole, produced by the α subunit, “locking” the TSα in the closed confirmation could block supplied labeled indole from entry. Such an inhibitory mechanism was first postulated based on the protein structures in the presence of aryl sulfonamides, also proposed as TSα inhibitors [56, 57]. These structures showed the α and β subunits in closed conformations with blocked access into the α and β sites in the presence of inhibitors. Interestingly, the inhibition pattern seen with Trp labeling is not recapitulated with IAA; I26 has little effect on the low level of [^13^C_6_]anthranilate labeling of IAA (Fig 9) and [^13^C_8_,^15^N_1_]indole labeling is reduced to near zero by I26 (Fig 10). This might seem in contrast with the data in Fig 6 which show significant [^13^C_8_,^15^N_1_]indole labeling of IAA when the predominate Trp pathway to IAA [49], the IPyA pathway, is blocked. However, there are several possible explanations for the data. First, TS could be involved in both Trp-dependent and Trp-independent IAA biosynthesis, although data from Trp auxotrophic mutants would suggest otherwise (reviewed in [58]). Indole-3-glycerol-phosphate (IGP) could be the precursor to IAA. Its formation by a reverse activity of any TSα-like enzyme would be expected to be inhibited by I26 as described for the forward reaction. Finally, an enzyme activity that forms a product important for IAA biosynthesis from indole could be a secondary target for inhibition by this allosteric mimic.

Stable isotope labeling followed by high resolution LC-MS/MS analysis is a powerful technique that can be used effectively to investigate labeling kinetics between multiple experimental groups and in tissues exposed to effectors of metabolic pathway activities. Determining relative changes to specific steps over short time intervals in a less well characterized metabolic pathway can aid in the understanding of how specific steps respond to disruptions, especially in steps catalyzed by multiple enzymes with similar and overlapping functions. These technologies can be easily adapted to measure processes in particular cellular fractions to determine in real-time cellular compartment dynamics. Similarly, stable isotopes allow the methods to be extended to be used in dynamic pulse-chase experimental designs followed by LC-MS/MS in order to investigate individual compound turnover.

## Supporting information

S1 Table Chemical inhibitors used for auxin biosynthetic pathway analysis. (PDF)

## References

[1] Li W, Zhou Y, Liu X, Yu P, Cohen JD, Meyerowitz EM. LEAFY controls auxin response pathways in floral primordium formation. Sci Signal. 2013;6(270):ra23. doi: 10.1126/scisignal.2003937 23572147 PMC4122305

[2] Ljung K, Hul AK, Kowalczyk M, Marchant A, Celenza J, Cohen JD, et al. Biosynthesis, conjugation, catabolism and homeostasis of indole-3-acetic acid in *Arabidopsis thaliana*. Plant Mol Biol. 2002;50(2):309–32. 12175022 10.1023/a:1016024017872

[3] Cohen JD, Gray WM. Auxin metabolism and signaling. Plant Hormone Signaling. 2006;24:37–66.

[4] Jiang Z, Li J, Qu LJ. Auxins. Hormone Metabolism and Signaling in Plants. 2017;39–76.

[5] Cooke TJ, Poli D, Sztein AE, Cohen JD. Evolutionary patterns in auxin action. In: Perrot-Rechenmann C, Hagen G, editors. Auxin Molecular Biology. New York: Springer Dordrecht; 2002. p. 319–38. 12036257

[6] Chokkathukalam A, Kim DH, Barrett MP, Breitling R, Creek DJ. Stable isotope-labeling studies in metabolomics: new insights into structure and dynamics of metabolic networks. Bioanalysis. 2014;6(4):511–24. doi: 10.4155/bio.13.348 24568354 PMC4048731

[7] Cooney TP, Nonhebel HM. Biosynthesis of indole-3-acetic acid in tomato shoots: Measurement, mass-spectral identification and incorporation of 2H from 2H2O into indole-3-acetic acid, D- and L-tryptophan, indole-3-pyruvate and tryptamine. Planta. 1991;184(3):368–76. doi: 10.1007/BF00195339 24194155

[8] Nishimura T, Nakano H, Hayashi K ichiro, Niwa C, Koshiba T. Differential downward stream of auxin synthesized at the tip has a key role in gravitropic curvature via TIR1/AFBs-mediated auxin signaling pathways. Plant Cell Physiol. 2009;50(11):1874–85. doi: 10.1093/pcp/pcp129 19897572

[9] Jones B, Gunnerås SA, Petersson SV, Tarkowski P, Graham N, May S, et al. Cytokinin regulation of auxin synthesis in *Arabidopsis* involves a homeostatic feedback loop regulated via auxin and cytokinin signal transduction. Plant Cell. 2010;22(9):2956–69. 20823193 10.1105/tpc.110.074856 PMC2965550

[10] Tivendale ND, Davies NW, Molesworth PP, Davidson SE, Smith JA, Lowe EK, et al. Reassessing the role of N-hydroxytryptamine in auxin biosynthesis. Plant Physiol. 2010;154(4):1957–65. doi: 10.1104/pp.110.165803 20974893 PMC2996026

[11] Nishimura T, Hayashi K, Suzuki H, Gyohda A, Takaoka C, Sakaguchi Y, et al. Yucasin is a potent inhibitor of YUCCA, a key enzyme in auxin biosynthesis. Plant J. 2014;77(3):352–66. doi: 10.1111/tpj.12399 24299123

[12] Ljung K, Hull AK, Celenza J, Yamada M, Estelle M, Normanly J, et al. Sites and regulation of auxin biosynthesis in Arabidopsis roots. Plant Cell. 2005;17(4):1090–104. doi: 10.1105/tpc.104.029272 15772288 PMC1087988

[13] Pieck M, Yuan Y, Godfrey J, Fisher C, Zolj S, Vaughan D, et al. Auxin and tryptophan homeostasis are facilitated by the ISS1/VAS1 aromatic aminotransferase in *Arabidopsis*. Genetics. 2015;201(1):185–99. 26163189 10.1534/genetics.115.180356 PMC4566262

[14] Glawischnig E, Tomas A, Eisenreich W, Spiteller P, Bacher A, Gierl A. Auxin biosynthesis in maize kernels. Plant Physiol. 2000;123(3):1109–20. doi: 10.1104/pp.123.3.1109 10889260 PMC59074

[15] Epstein E, Cohen JD, Slovin JP. The biosynthetic pathway for indole-3-acetic acid changes during tomato fruit development. Plant Growth Regul. 2002;38(1):15–20.

[16] Wang B, Chu J, Yu T, Xu Q, Sun X, Yuan J, et al. Tryptophan-independent auxin biosynthesis contributes to early embryogenesis in Arabidopsis. Proc National Acad Sci. 2015;112(15):4821–6. doi: 10.1073/pnas.1503998112 25831515 PMC4403211

[17] Kramer EM, Ackelsberg EM. Auxin metabolism rates and implications for plant development. Front Plant Sci. 2015;6:150. doi: 10.3389/fpls.2015.00150 25852709 PMC4362085

[18] Tam YY, Slovin JP, Cohen JD. Selection and characterization of alpha-methyltryptophan resistant lines of *Lemna gibba* showing a rapid rate of indole-3-acetic acid turnover. Plant Physiol. 1995;107(1):77–85. 12228344 10.1104/pp.107.1.77 PMC161170

[19] Rapparini F, Tam YY, Cohen JD, Slovin JP. Indole-3-acetic acid metabolism in *Lemna gibba* undergoes dynamic changes in response to growth temperature. Plant Physiol. 2002;128(4):1410–6. 11950989 10.1104/pp.011005 PMC154268

[20] Tillmann M, Tang Q, Cohen JD. Protocol: analytical methods for visualizing the indolic precursor network leading to auxin biosynthesis. Plant Methods. 2021;17(1):63. doi: 10.1186/s13007-021-00763-0 34158074 PMC8220744

[21] Lincoln C, Britton JH, Estelle M. Growth and development of the *axr1* mutants of *Arabidopsis*. Plant Cell. 1990;2(11):1071–80. 1983791 10.1105/tpc.2.11.1071 PMC159955

[22] Liu X, Hegeman AD, Gardner G, Cohen JD. Protocol: high-throughput and quantitative assays of auxin and auxin precursors from minute tissue samples. Plant Methods. 2012;8(1):1–17. 22883136 10.1186/1746-4811-8-31 PMC3457856

[23] Tsugafune S, Mashiguchi K, Fukui K, Takebayashi Y, Nishimura T, Sakai T, et al. Yucasin DF, a potent and persistent inhibitor of auxin biosynthesis in plants. Sci Rep-uk. 2017;7(1):13992. doi: 10.1038/s41598-017-14332-w 29070794 PMC5656596

[24] Narukawa‐Nara M, Nakamura A, Kikuzato K, Kakei Y, Sato A, Mitani Y, et al. Aminooxy‐naphthylpropionic acid and its derivatives are inhibitors of auxin biosynthesis targeting L‐tryptophan aminotransferase: structure–activity relationships. Plant J. 2016;87(3):245–57. doi: 10.1111/tpj.13197 27147230

[25] Finn J, Langevine C, Birk I, Birk J, Nickerson K, Rodaway S. Rational herbicide design by inhibition of tryptophan biosynthesis. Bioorganic Med Chem Lett. 1999;9(16):2297–302. doi: 10.1016/s0960-894x(99)00340-6 10476857

[26] Dias MVB, Canduri F, da Silveira NJF, Czekster CM, Basso LA, Palma MS, et al. Molecular models of tryptophan synthase from *Mycobacterium tuberculosis* complexed with inhibitors. Cell Biochem Biophys. 2006;44(3):375–84. 16679524 10.1385/CBB:44:3:375

[27] Magnus V, Bandurski RS, Schulze A. Synthesis of 4,5,6,7 and 2,4,5,6,7 Deuterium-labeled Indole-3-Acetic Acid for Use in Mass Spectrometric Assays. Plant Physiol. 1980;66(4):775–81. doi: 10.1104/pp.66.4.775 16661520 PMC440721

[28] Bloch K, Anker H. An extension of the isotope dilution method. Science. 1948;107(2774):228. doi: 10.1126/science.107.2774.228 17749210

[29] Ahmad A, Eelnurme I, Spenser ID. Indolyl-3-acetaldoxime. Can J Chem. 1960;38(12):2523–2523.

[30] Barkawi LS, Tam YY, Tillman JA, Normanly J, Cohen JD. A high-throughput method for the quantitative analysis of auxins. Nat Protoc. 2010;5(10):1609–18. doi: 10.1038/nprot.2010.118 20885372

[31] Chambers MC, Maclean B, Burke R, Amodei D, Ruderman DL, Neumann S, et al. A cross-platform toolkit for mass spectrometry and proteomics. Nat Biotechnol. 2012;30(10):918–20. doi: 10.1038/nbt.2377 23051804 PMC3471674

[32] Evans EM, Freund DM, Sondervan VM, Cohen JD, Hegeman AD. Metabolic patterns in Spirodela polyrhiza revealed by 15N stable isotope labeling of amino acids in photoautotrophic, heterotrophic, and mixotrophic growth conditions. Front Chem. 2018;6:191. doi: 10.3389/fchem.2018.00191 29904627 PMC5990592

[33] Fan KT, Rendahl AK, Chen WP, Freund DM, Gray WM, Cohen JD, et al. Proteome scale-protein turnover analysis using high resolution mass spectrometric data from stable-isotope labeled plants. J Proteome Res. 2016;15(3):851–67. doi: 10.1021/acs.jproteome.5b00772 26824330 PMC5482238

[34] Smith CA, Want EJ, O’Maille G, Abagyan R, Siuzdak G. XCMS: processing mass spectrometry data for metabolite profiling using nonlinear peak alignment, matching, and identification. Anal Chem. 2006;78(3):779–87. doi: 10.1021/ac051437y 16448051

[35] Huttlin EL, Hegeman AD, Harms AC, Sussman MR. Comparison of full versus partial metabolic labeling for quantitative proteomics analysis in *Arabidopsis thaliana*. Mol Cell Proteomics. 2007;6(5):860–81. 17293592 10.1074/mcp.M600347-MCP200

[36] Cohen JD, Baldi BG, Slovin JP. C(6)-[benzene ring]-indole-3-acetic Acid: a new internal standard for quantitative mass spectral analysis of indole-3-acetic Acid in plants. Plant Physiol. 1986;80(1):14–9. doi: 10.1104/pp.80.1.14 16664570 PMC1075048

[37] Bateman RJ, West T, Yarasheski K, Patterson BW, Lucey B, Cirrito JR, et al. Chapter 11—Stable isotope labeling kinetics in CNS translational medicine: introduction to SILK technology. Handb Behav Neurosci. 2019;29:173–90.

[38] Jin H, Moseley HNB. Hierarchical harmonization of atom-resolved metabolic reactions across metabolic databases. Metabolites. 2021;11(7):431. doi: 10.3390/metabo11070431 34209357 PMC8307411

[39] Bartel B, LeClere S, Magidin M, Zolman BK. Inputs to the active indole-3-acetic acid pool: de novo synthesis, conjugate hydrolysis, and indole-3-butyric acid β-oxidation. J Plant Growth Regul. 2001;20(3):198–216.

[40] Pengelly WL, Bandurski RS. Analysis of indole-3-acetic acid metabolism in *Zea mays* using deuterium oxide as a tracer. Plant Physiol. 1983;73(2):445–9. 16663236 10.1104/pp.73.2.445 PMC1066481

[41] Wright AD, Sampson MB, Neuffer MG, Michalczuk L, Slovin JP, Cohen JD. Indole-3-acetic acid biosynthesis in the mutant maize orange pericarp, a tryptophan auxotroph. Science. 1991;254(5034):998–1000. doi: 10.1126/science.254.5034.998 17731524

[42] Ribaut JM, Schaerer S, Pilet PE. Deuterium-labelled indole-3-acetic acid neo-synthesis in plantlets and excised roots of maize. Planta. 1993;189(1):80–2.

[43] Jensen PJ, Bandurski RS. Incorporation of deuterium into indole-3-acetic acid and tryptophan in *Zea mays* seedlings grown on 30% deuterium oxide. J Plant Physiol. 1996;147(6):697–702.

[44] Bialek K, Michalczuk L, Cohen JD. Auxin biosynthesis during seed germination in *Phaseolus vulgaris*. Plant Physiol. 1992;100(1):509–17. 16652991 10.1104/pp.100.1.509 PMC1075579

[45] Yang XY, Chen WP, Rendahl AK, Hegeman AD, Gray WM, Cohen JD. Measuring the turnover rates of Arabidopsis proteins using deuterium oxide: an auxin signaling case study. Plant J Cell Mol Biology. 2010;63(4):680–95. doi: 10.1111/j.1365-313X.2010.04266.x 20525007

[46] Smith RM. Important Mass Spectral Rearrangements. In: Smith RM, editor. Understanding Mass Spectra: A Basic Approach, Second Edition. 2004. p. 207–37.

[47] Müntz K, Becker C, Pancke J, Schlereth A, Fischer J, Horstmann C, et al. Protein degradation and nitrogen supply during germination and seedling growth of vetch (Vicia sativa L.). J Plant Physiol. 1998;152(6):683–91.

[48] Nonhebel H, Cooney T, Simpson R. The route, control and compartmentation of auxin synthesis. Funct Plant Biol. 1993;20(5):527.

[49] Tillmann M, Tang Q, Gardner G, Cohen JD. Complexity of the auxin biosynthetic network in Arabidopsis hypocotyls is revealed by multiple stable-labeled precursors. Phytochemistry. 2022;200:113219. doi: 10.1016/j.phytochem.2022.113219 35523282

[50] Erb M, Kliebenstein DJ. Plant secondary metabolites as defenses, regulators, and primary metabolites: the blurred functional trichotomy. Plant Physiol. 2020;184(1):39–52. doi: 10.1104/pp.20.00433 32636341 PMC7479915

[51] Zhang R, Wang B, Ouyang J, Li J, Wang Y. *Arabidopsis* indole synthase, a homolog of tryptophan synthase alpha, is an enzyme involved in the Trp-independent indole-containing metabolite biosynthesis. J Integr Plant Biol. 2008;50(9):1070–7. 18844775 10.1111/j.1744-7909.2008.00729.x

[52] Yin R, Frey M, Gierl A, Glawischnig E. Plants contain two distinct classes of functional tryptophan synthase beta proteins. Phytochemistry. 2010;71(14–15):1667–72. doi: 10.1016/j.phytochem.2010.07.006 20701934

[53] Liu C, Sun Q, Zhao L, Li Z, Peng Z, Zhang J. Heterologous expression of the transcription factor EsNAC1 in Arabidopsis enhances abiotic stress resistance and retards growth by regulating the expression of different target genes. Front Plant Sci. 2018;9:1495. doi: 10.3389/fpls.2018.01495 30374363 PMC6196249

[54] Nonhebel H. Tryptophan-independent indole-3-acetic acid synthesis: Critical evaluation of the evidence. Plant Physiol. 2015;169(2):1001–5. doi: 10.1104/pp.15.01091 26251310 PMC4587473

[55] Michalska K, Chang C, Maltseva NI, Jedrzejczak R, Robertson GT, Gusovsky F, et al. Allosteric inhibitors of *Mycobacterium tuberculosis* tryptophan synthase. Protein Sci. 2020;29(3):779–88. 31930594 10.1002/pro.3825 PMC7020977

[56] Hilario E, Caulkins BG, Huang YMM, You W, Chang CEA, Mueller LJ, et al. Visualizing the tunnel in tryptophan synthase with crystallography: Insights into a selective filter for accommodating indole and rejecting water. Biochimica Et Biophysica Acta Bba—Proteins Proteom. 2016;1864(3):268–79. doi: 10.1016/j.bbapap.2015.12.006 26708480 PMC4732270

[57] Ngo H, Kimmich N, Harris R, Niks D, Blumenstein L, Kulik V, et al. Allosteric regulation of substrate channeling in tryptophan synthase: modulation of the L-serine reaction in stage I of the beta-reaction by alpha-site ligands. Biochemistry-us. 2007;46(26):7740–53. 10.1021/bi7003872 17559232

[58] Tivendale ND, Ross JJ, Cohen JD. The shifting paradigms of auxin biosynthesis. Trends Plant Sci. 2014;19(1):44–51. doi: 10.1016/j.tplants.2013.09.012 24524164

