# Supplementary material for: Analytical methods for stable isotope labeling to elucidate rapid auxin kinetics in Arabidopsis thaliana
Source: PLoS One. 2024 May 22;19(5):e0303992. doi: 10.1371/journal.pone.0303992 (PMC11111016; doi:10.1371/journal.pone.0303992)
Supplement: S1 Table — (PDF) [file pone.0303992.s001.pdf]

S1 Table. Chemical inhibitors used for auxin biosynthetic pathway analysis

Inhibitor name	Representative structure(s)	Chemical structures	Target	Mode of action
**Yucasin DF (YDF)**	5-[2,6-difluorophenyl]-2,4-dihydro-[1,2,4]-triazole-3-thione	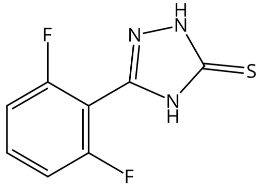	YUCCA	Competitive inhibitor
**Pyruvamines (PVM) “Type II compounds” (Derivatives of Type I compounds)**	PVM2153; Benzene propanoic acid, 3,4-dichloro-α-[(1,3-dihydro-1,3-dioxo-2H-isoindol-2-yl)oxy]-methyl ester	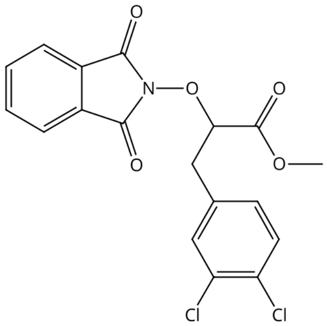	TAA1	Competitive inhibitor
**Arylsulfide phosphonates**	I26; RHA41161; [4-[(2-amino phenyl)sulfanyl] butyl]phosphonic acid	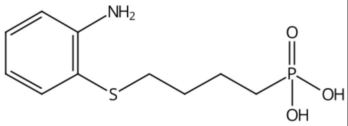	Trp synthase α	Transition state analog
